# Development and evaluation of time-resolved rapid immunofluorescence test for detection of TSOL18 specific antibody in porcine cysticercosis infections

**DOI:** 10.1186/s12917-024-04034-7

**Published:** 2024-05-08

**Authors:** Dejia Zhang, Rui Duan, Jing Liu, Mengqi Wang, Yi Yang, Yongjun Zhao, Mingyuan Liu, Shumin Sun

**Affiliations:** 1 College of Veterinary Medicine, Yunnan Agricultural University, Kunming, 650201 China; 2 College of Animal Science and Technology, Inner Mongolia MinZu University, Tongliao, 028000 China; 3 Qingdao Special Servicemen Recuperation Center of PLA Navy, Qingdao, 266071 China; 4 State Key Laboratory for Diagnosis and Treatment of Severe Zoonotic Infectious Diseases, Key Laboratory for Zoonosis Research of the Ministry of Education, Institute of Zoonosis, and College of Veterinary Medicine, Jilin University, Changchun, 130062 China; 5 Jiangsu Co-innovation Center for Prevention and Control of Important Animal Infectious Diseases and Zoonoses, Yangzhou, 225000 China

**Keywords:** *Cysticercus cellulosae*, Time-resolved fluorescent microspheres, Immunochromatographic strip, Immunodiagnosis

## Abstract

**Background:**

Porcine cysticercosis, a serious zoonotic parasitic disease, is caused by the larvae of *Taenia solium* and has been acknowledged by the World Organization for Animal Health. The current detection methods of *Cysticercus cellulosae* cannot meet the needs of large-scale and rapid detection in the field. We hypothesized that the immunofluorescence chromatography test strip (ICS) for detecting *Cysticercus cellulosae*, according to optimization of a series of reaction systems was conducted, and sensitivity, specificity, and stability testing, and was finally compared with ELISA. This method utilizes Eu^3+^-labeled time-resolved fluorescent microspheres (TRFM) coupled with TSOL18 antigen to detect TSOL18 antibodies in infected pig sera.

**Results:**

ICS and autopsy have highly consistent diagnostic results (*n* = 133), as determined by Cohen’s κ analysis (κ = 0.925). And the results showed that the proposed ICS are high sensitivity (0.9459) with specificity (0.9792). The ICS was unable to detect positive samples of other parasites. It can be stored for at least six months at 4℃.

**Conclusions:**

In summary, we established a TRFM-ICS method with higher sensitivity and specificity than indirect ELISA. Results obtained from serum samples can be read within 10 min, indicating a rapid, user-friendly test suitable for large-scale field detection.

**Supplementary Information:**

The online version contains supplementary material available at 10.1186/s12917-024-04034-7.

## Introduction

*Cysticercus cellulosae*, the metacestode stage of *Taenia solium*, causes cysticercosis, which is a serious zoonotic parasitic disease and has been listed by the World Health Organization as one of the neglected tropical diseases [1]. The Chinese List of Zoonotic Infectious Diseases still includes cysticercosis as of June 2022. *Taenia solium* exists throughout America [2] and sub-Saharan Africa [3], Bali and Papua in Indonesia [4] and Asia [5]. The prevalence rates of porcine cysticercosis in Africa, Latin America, and Asia were 17.37%, 13.03%, and 15.68% respectively between 1989 and 2014 [5]. However, Uganda [6], Ghana [7], Cameroon [8] in Africa, and Cambodia [9], have been plagued by the porcine cysticercosis in the past decade, with prevalence rates reaching 4.8%, 24.9%, 24.8%, and 4.7% respectively.

The symptoms caused by cysticercosis are related to its parasitic location and quantity, but the most concerned is neurocysticercosis [5]. 1/3 of epilepsy was caused by neurocysticercosis in low-and middle-income countries [10]. Secondly, ocular cysticercosis is also a serious clinical problem. Most of the parasitic cysticercosis are located in the deep part of the eyeball, and vitreous cysts may hinder vision or even blindness [11]. Cysticercosis is also a serious threat to the health of pigs. It can lead to disability or death in severe cases.

A rapid detection method with high accuracy, specificity, and sensitivity can reduce the global burden of cysticercosis, especially in remote areas of many developing countries that lack good medical facilities. If the inspection during slaughtering and quarantine mainly relies on identifying *Trichinella spiralis* and Cysticercus cellulosae by the naked eye, it can easily cause missed detection, seriously affecting the meat quality and posing a potential often needs to be assessed in cysticercosis-endemic areas [12]. The current common detection methods are indirect enzyme-linked immunosorbent assay (Indirect ELISA) and dot avidin-biotin complex enzyme-linked immunosorbent assay (Dot-ABC-ELISA) [13, 14]. The basic principle of ELISA technology can be traced back to 1941, and it is still one of the most commonly used methods in the laboratory [15]. However, the method is not suitable forrapid detection in the field.

At present, there are a variety of immunochromatographic methods on the market. The test strips based on gold nanoparticles are very common in the market, but different batches of products vary greatly [16]. Moreover, the chromaticity and colloidal stability of gold nanoparticles are suboptimal, resulting in diminished sensitivity of the colloidal gold test strip [16]. One of the main drawbacks of traditional fluorescence quantitative analysis is background interference [17]. Time-resolved fluorescence microsphere immunochromatography strip test (TRFM-ICST) is a relatively novel detection technique. The time-resolved lanthanide chelated microspheres have a longer fluorescence signal lifetime, with a long decay time of 10-2000 µs, about 10^3^ times that of traditional fluorescent compared with traditional fluorescent microspheres [18, 19]. The fluorescence signal of TRFM can still be measured after a certain excitation time interval, which avoids the influence of background fluorescence signal [18, 19]. However, TRFM-ICST can be read under a 365 nm wavelength UV light and analyzed through an immunoassay analyzer. The TRFM can wrap thousands of fluorescent molecules greatly increasing the labeling efficiency [20]. TRFM contains carboxyl groups with appropriate density, which can be used for covalently coupling of proteins or antibodies to improve the stability of the labeling [20]. Moreover, TRFM-ICST offers a series of advantages, including high specificity, sensitivity and stability, wide linear range, multi-marker detection, etc [18–20].

In the life cycle of *Taenia solium*, the oncosphere stage is considered to be the key period for invading the host, which generates specific antigen called TSOL18 with strong immunogenicity and antigenicity [11]. The TSOL18 gene has been widely used as a potential candidate gene in oncospheres vaccines, owing to the good immunogenicity of the TSOL18 protein [11]. Pigs attained a nearly complete immune protection rate of 99.5% when they were immunized solely with the recombinant TSOL18 antigen [21]. A research used TSOL18 to develop a method for detecting porcine cysticercosis based on a lateral-flow assay (LFA) that uses up-converting phosphor technology [22]. The sensitivity of this method was 93.59% and the specificity was 100% [22]. In this study, we aimed to assess a simple, rapid, and stable TRFM-ICS using TSOL18 antigen to achieve large-scale and rapid field detection of *Cysticercus cellulose*.

## Materials and methods

### Materials

Sartorius (USA) provided the nitrocellulose (NC) membrane (CN140). Mouse anti-pig IgG antibodies and rabbit anti-goat IgG antibodies were obtained from Beijing Biolab Technology (China). Carboxyl group-modified europium nanoparticles (EuNPs) were purchased from Thermo Fisher (USA). 1-(3-dimethylaminopropyl)-3-ethyl carbodiimide (EDC) was purchased from Tokyo Chemical Industry (China). Bovine serum albumin (BSA) and Tween-20 were obtained from Solarbio (China). N-hydroxysuccinimide (NHS) was purchased from Sigma Aldrich (Japan). 2-morpholine ethanesulfonic acid (MES) was purchased from Genview (China). Proclin300 was obtained from Yuanye Biology (China). Jinbiao Biotech (China) provided the samples, conjugates, absorbents, and plastic backing. Goat anti-rabbit IgG antibodies were obtained from Cell Signal Technology (USA).The TSOL18 Recombinant protein was synthesized by Sangon Biotech (China) [23].

The UV lamp was purchased from Shenzhen Feike Technology (China). The fluorescence quantitative analyzer was from Weice Biotech (China). The XYZ3060 dispenser was obtained from BioDot (USA) and XYZ three-dimensional gold spraying instrument HM3230 was from Jinbiao Biotech (China). The XM-P15H ultrasonic cleaner was purchased from Xiaomei ultrasonic instrument (China).

### Ethical approval

This study was approved by the ethics committee of Inner Mongolia MinZu University (approval no.IMUN20190301). We certify that the study was performed in accordance with the 1964 declaration of HELSINKI and later amendments.

### Study animals and serum samples

A total of 105 suspected pigs were purchased from farmers suspected of being infected with cysticercosis in several areas of Inner Mongolia between 2014 and 2018 (Clinical symptoms include stunted growth and development of pigs, stiffness of the forelimbs, and granular solids in the subcutaneous muscles of the medial thigh. A few pigs have symptoms of epilepsy). Our sample selection principle is: natural infection, no major diseases, and the possibility of cross infection with other parasites should be excluded as much as possible. None of the sampled pigs had been vaccinated against cysticercosis. Positive and negative samples should be ensured to live in the same environment, and the sample size should not be too small and sufficient for convincing analysis.

Blood samples were obtained by jugular vein puncture, and stool samples were obtained from rectum. The serum samples of these pigs were preserved at Inner Mongolia Minzu University’s Clinical Laboratory of Animal Science and Technology, China.

A total of 69 porcine sera infected with *Taenia hydatigena* (5), *Toxoplasma gondii* (3), *Trichinella spiralis* (20), *Taenia asiatica* (4), *Ascaris suum* (9) and negative pig serum samples without any infection (28 samples) were provided by the State Key Laboratory for Diagnosis and Treatment of Severe Zoonotic Infectious Diseases, Jilin University (China).

### Pig necropsies

We euthanized pigs by intravenous injection of excessive pentobarbital sodium (100 mg/kg). Pigs were carefully examined for the presence of cysts in the entrails and muscle tissues during the necropsy. The suspicious area was dissected into 1 cm thick slices using a surgical scalpel, and a thorough examination was conducted to identify the presence of *Cysticercus cellulosae*. The mature *Cysticercus cellulosae* exhibits an elongated oval shape, measuring approximately 6–10 mm in length and 5 mm in width. It is translucent in appearance, with a liquid-filled capsule. Additionally, it contains a white scolex, comparable in size to a millet seed. The parasite found within the brain exhibits a spherical shape, measuring 8–10 mm in diameter. Cysticercosis burden was classified as follows: negative (no cysts), mild if one to 20 cysts were found in the whole carcass, including the brain; moderate for 20 to 200 cysts; and severe for those with more than 200 cysts in the whole pig [24]. Overall a pig was considered positive if at least one cyst was found in the whole carcass.

In addition, we paid extra attention to the infection of other parasites (such as *Taenia hydatigena* and *Taenia asiatica*) in pigs’ entrails and muscle tissues during the necropsy.

### Gastrointestinal parasite identification and counts

Put 5–10 g of feces in a beaker, add 10–20 times the volume of saturated saline solution, and stir the feces until they are dissolved. Use 3–4 layers of gauze or copper mesh to filter the mixture. Observe and count the sample under the microscope after standing for 15 min [6].

### Method establishment based on enzyme-linked immunosorbent assay

The TSOL18 protein was diluted to 1 µg/mL using a carbonate buffer solution coating solution. Then, 100 µl antigen was added to each well of a 96-well ELISA plate and incubated at 37 °C for 2 h. Then 150 µl PBST buffer (PBS containing 0.05% Tween-20) was added to each well for washing and the plate was put on a shaker for 5 min × 5 times. Each hole were filled with 150 µl blocking solution (PBST containing 1% BSA) at 25 °C for 1.5 h. Each well was added with 100 µl blocking solution containing the primary antibody at 25℃ for 1.5 h after washed the plate. The not-infected or blank control pig serum was used to set the negative control group (verified using the gold standard method with a sample size of at least 5). Each well was added with 100 µl blocking solution containing the goat anti-porcine IgG secondary antibody (1:4000) at 37℃ for 0.5 h after washed the plate. In the darkness, 100 µl TMB was added to each well at 37℃ for 15 min after washed the plate. Then, 50 µl 2 mol/L H_2_SO_4_ was added to each well to stop the reaction. Finally, the plate was read for sample absorption at 450 nm by the enzyme labeling instrument and the results were examined. The sample was deemed positive if the OD value exceeded 2.1 times over the negative control sample.

### Coupling of time-resolved fluorescent microspheres with the antigen and antibodies

First, 10 µl of microspheres were centrifuged at 14,000×g for 20 min, and the supernatant was discarded. Then microspheres were resuspended in 100 µl (50 mM/L, pH 6.0) of MES, and the supernatant was again removed by centrifugation at 14,000×g for 20 min at 4℃. The recovered microspheres were incubated with 0.5 µl of EDC (10 mg/mL), 1.5 µl of NHS (15 mg/mL), and 100 µl of MES for 0.5 h. Afterward, the mixture was centrifuged for 20 min at 14,000×g to remove the supernatant. Next, the processed microspheres were resuspended in 200 µL of PB buffer (0.025 mol/L, pH 7.2) and subjected to ultrasonication for 6 min (300 W). Next, 6.7 µg of TSOL18 antigen was added and the mixture was incubated at room temperature (25 ± 5℃) for 3 h, followed by centrifugation at 14,000×g for 20 min to remove the supernatant. Next, the recovered pellet was added with 200 µl of 1% BSA and 0.1 M Glycine and blocking was performed for 2 h at room temperature (RT). The mixture was again centrifuged to remove the supernatant and the pellet was added with fluorescent probes dissolved in a preserved buffer (25 mM/L Tris containing 0.05% Tween-20, 0.15 mM/L NaCl, 0.05% Pro Clin 300, 1% BSA, and 5% Trehalose). Similar procedures were followed for the microspheres conjugation with goat anti-rabbit IgG [25].

### Composition of the immunofluorescence chromatography test strip (ICS)

The ICS is composed of a sample pad, conjugated pad, NC film, absorbent pad, and bottom plate. The fluorescent probes of TRFM-TSOL18 (capture probe) and TRFM- Goat anti-rabbit IgG (indicator probe) were mixed in a 4:1 ratio and sprayed on the conjugated pad with XYZ3060 dispensing machine. Similarly, mouse anti-pig IgG antibodies (2 mg/mL) were coated on NC membrane to make T-line for sample detection. Rabbit anti-goat IgG antibodies (1 mg/mL) were coated on the NC membrane to make a C-line for quality control. After drying at RT for 3 h, the five parts were assembled in the direction of chromatography, cut into the width of 4 mm, and stored at 4 ℃ [26].

### ICS detection principle

**Fig. 1:**
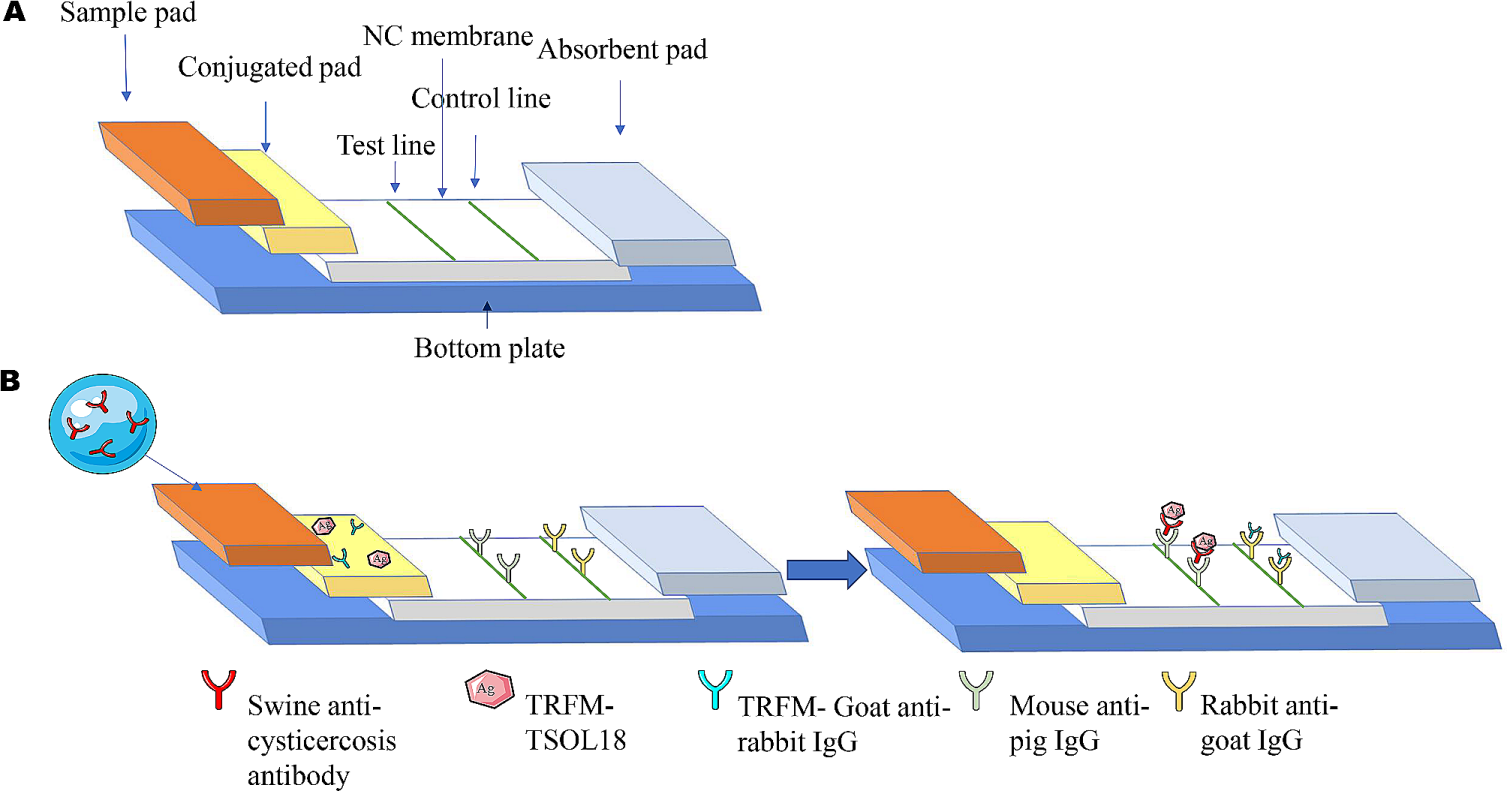
Schematic diagram of immunofluorescence chromatographic test strip for cysticercosis detection. **(A)** Assembly design of immunochromatographic strips. **(B)** Schematic diagram showing the chromatographic movement of constituents in the immunofluorescence chromatography test strip

**Fig. 2:**
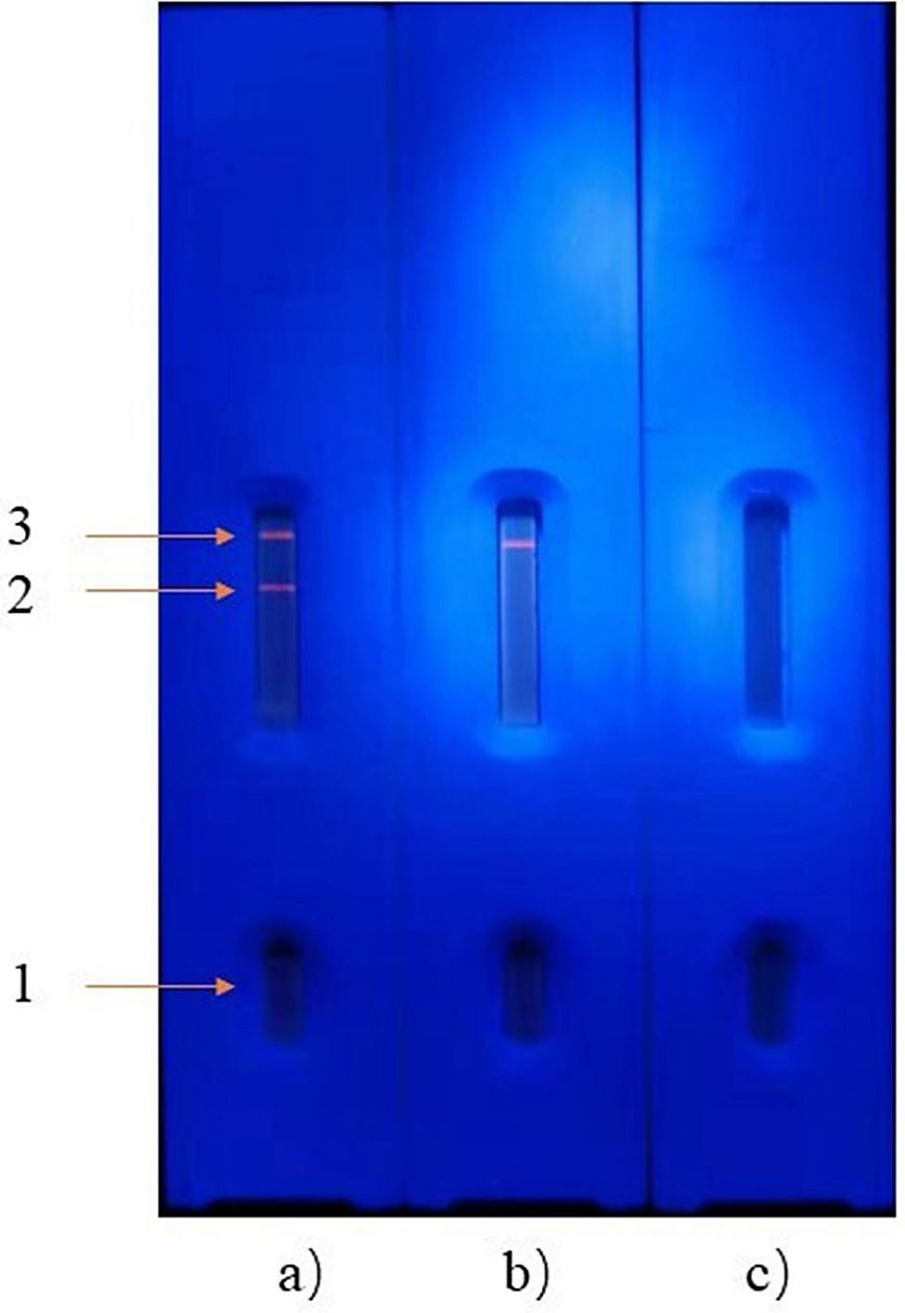
Positive and negative serum samples were detected under a UV lamp using ICS. 1: sample hole; 2: detection line; 3: quality control line. **(a)** is a schematic diagram of positive results; **(b)** is a schematic diagram of negative results; **(c)** is a schematic diagram of invalid results

The ICS principle of cysticercosis detection is illustrated in Fig. 1. The anti-*Cysticercus cellulosae* antibodies in serum samples are bind to the fluorescent microsphere-TSOL18 probe on the binding pad if detecting a positive sample. The fluorescent conjugate is subsequently captured by the mouse anti-porcine IgG at the T-line as it undergoes chromatographic movement. Similarly, the fluorescent microsphere-goat anti-rabbit IgG probe binds to rabbit anti-goat IgG on the C-line [26]. Finally, there are fluorescence signals at both the C and T lines.Only rabbit anti-goat IgG will bind with the fluorescent microsphere goat anti-rabbit IgG probe at the C-line when detecting negative serum (Fig. 2).

### The cut off value for the ICS

The fluorescence signal values of the T line were read after detecting the serum samples from 80 parasite-free pigs using the ICS. The cut off value was determined through the analysis. Samples with T line values lower than the cut off value were considered negative, whereas those equal to or higher than the cut off value were considered positive.

### Optimization of ICS

The sample pad, generally made of cellulose or glass fiber, can transport the sample to other parts of the test strip. Its functions include separating sample components, removing interference, and adjusting the pH. Firstly, the sample gasket materials (SB08, 8964, XQY8, ST17, SB06, RB45, 6613, and RB65) and made of glass fiber and polyester fiber were evaluated for their applicability in ICS. Secondly, TSOL18 and fluorescent microspheres coupled at 1:5, 1:10, 1:15, and 1:20 were evaluated [25].

### Analytical sensitivity, and specificity, and stability of ICS

The serum samples from pigs infected with *Cysticercus cellulosae* were tested for dilution at 1:25, 1:50, 1:100, 1:200, 1:400, 1:800, 1:1600, and 1:3200 by ICS. Thereafter, the results of the serologically positive samples were compared with results obtained from ELISA and assessment of autopsies.The serum samples infected with *Toxoplasma gondii*, *Trichinella spiralis*, *Taenia asiatica*, *Taenia hydatigena*, and *Ascaris suum* were detected by TRFM-ICST to evaluate analytical specificity of the ICS.

The same batch of negative (10) and positive serums (10) was tested at different time points and two temperatures (4 and 25 ℃) and each test had three replicates. The results at different time points were compared with the initial results to calculate the relative deviation: a relative deviation of less than 15% was accepted [22].

### Data analysis

The ICS data from the fluorescence reader and that of T-line fluorescence were imported into GraphPad Prism8 software, and the results are expressed as means ± SD. SPSS 24 statistical software was used for statistical analysis.

## Result

### Necropsy results

**Fig. 3:**
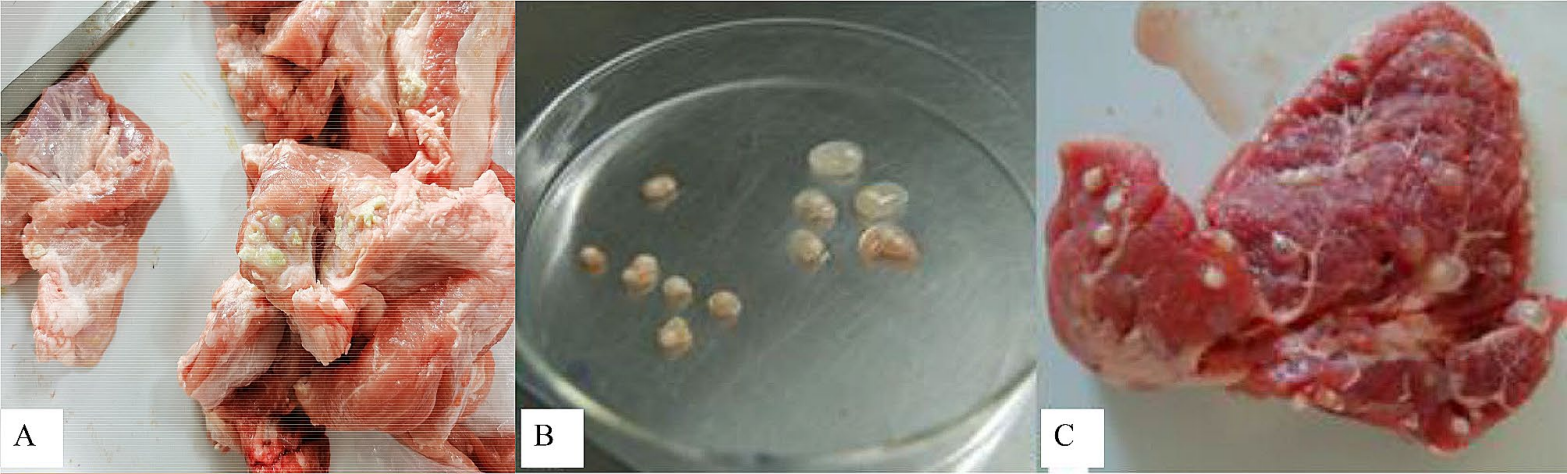
Autopsy pictures (partial). **(A)** Caseous (inactive) cysticerci. **(B) ***Cysticercus cellulosae*. **(C)** Cysts (active)

A total of 105 suspicious positive pigs were purchased, from which 64 were > 9 months old, and 41 were ≤ 9 months. The 37 pigs (35.2%) had porcine cysticercosis, whereas 68 pigs (64.8%) were free from it. Among these 37 infected pigs, 7, 26 and 4 had mild, moderate and severe infection burdens (Fig. 3).

No infections of *Taenia hydatigena*, *Taenia asiatica*, and *Trichinella spiralis* were found during the autopsy of all pigs.

### Porcine rectal stool examination results

No parasite eggs were found. This observation indicates the absence of cross-infection of parasites of coccidia, *Ascaris spp.* and *Trichuris spp.* in pigs.

### ICS cut‑off value

**Fig. 4:**
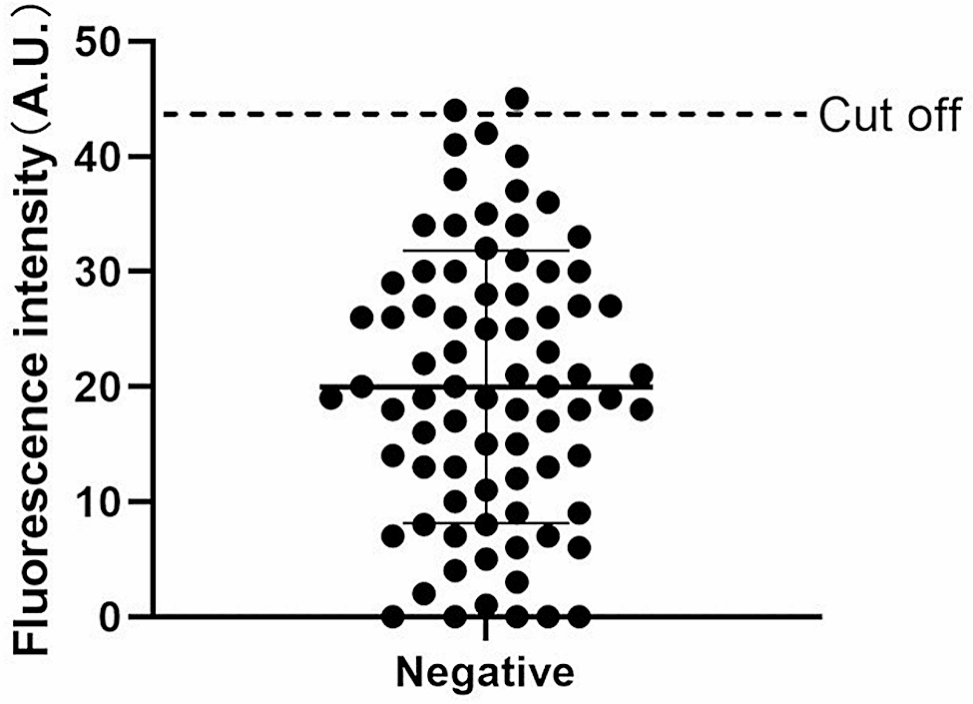
Distribution of negative results to determine the cut-off value of ICS

Negative serum samples from 80 pigs without parasite infection were detected by ICS. We used Probability-probability Plot and Quantile-quantile Plot to test all the T-line fluorescence values of ICS after inputting them into the SPSS analysis software. It was found that the data conformed to the normal distribution. We set the confidence level at 95% and use x̅±2SD to establish the confidence intervals. The cut-off value of TRFM-TSOL18-ICS based on x̅±2SD is 43.63 (Fig. 4).

### ICS optimization

**Fig. 5:**
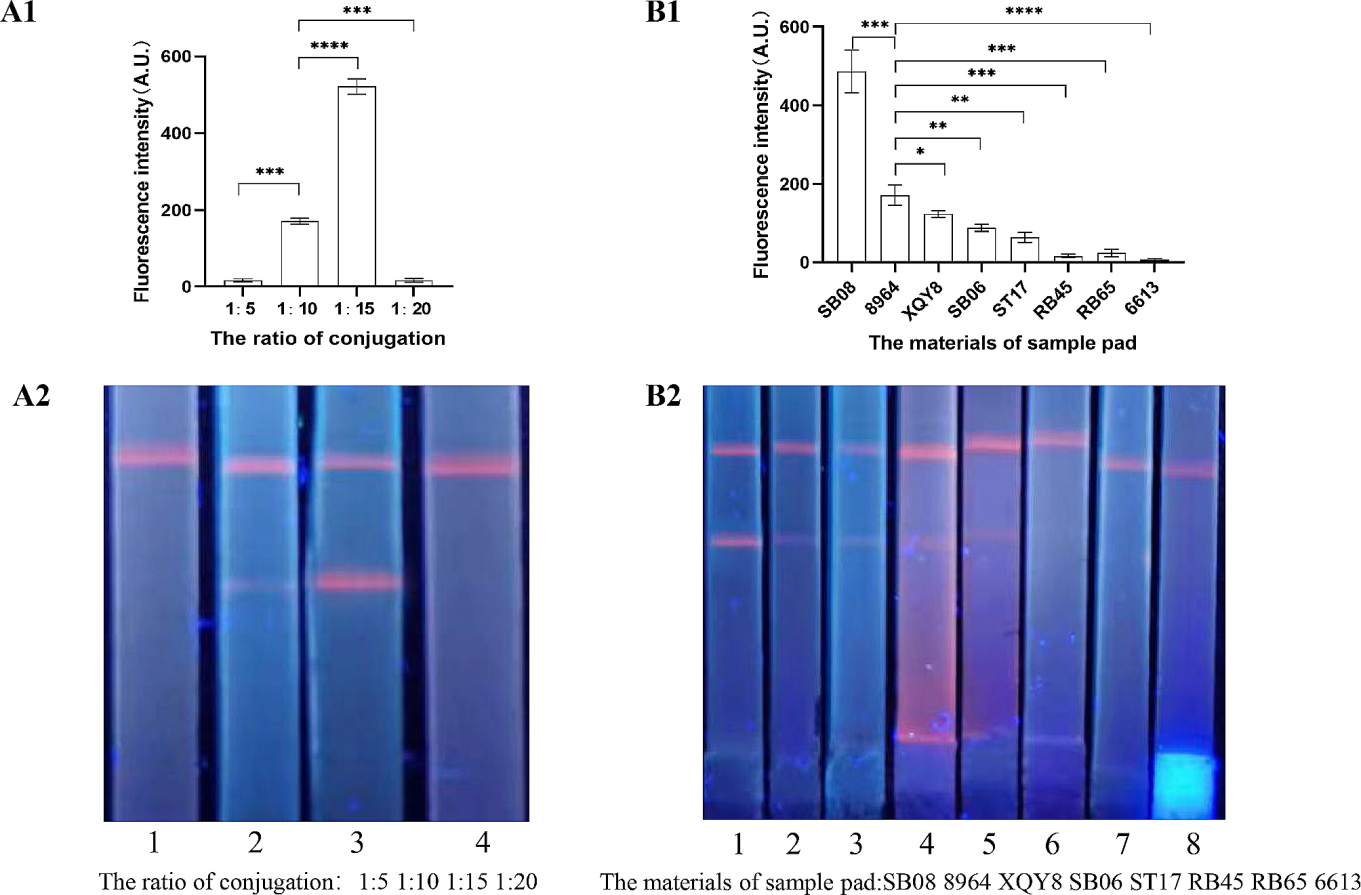
Optimization of ICS. **(A)** Rate of fluorescent particle conjugation with TSOL18 antigens: lanes 1 to 4 denote immunofluorescent microsphere-TSOL18 antigen ratio of 1:5, 1:10, 1:15, and 1:20, respectively. **(B)** Optimization of sample pad materials: lanes 1 to 8 are SB08, 8964, XQY8, SB06, ST17, RB45, RB65, and 6613, respectively. *P < 0.05, **P < 0.01, ***P < 0.001,****P < 0.0001

The sample pad materials and the ratio of time immunofluorescence resolved microsphere antigen in ICS were optimized for a bright fluorescence signal at the T-line. SB08 sample pads produced a stronger fluorescence signal at the T line. Also, the immunofluorescent microsphere- TSOL18 antigen ratio of 1:15 was found the best for a stronger fluorescence signal (Fig. 5).

### Analytical sensitivity test

**Fig. 6:**
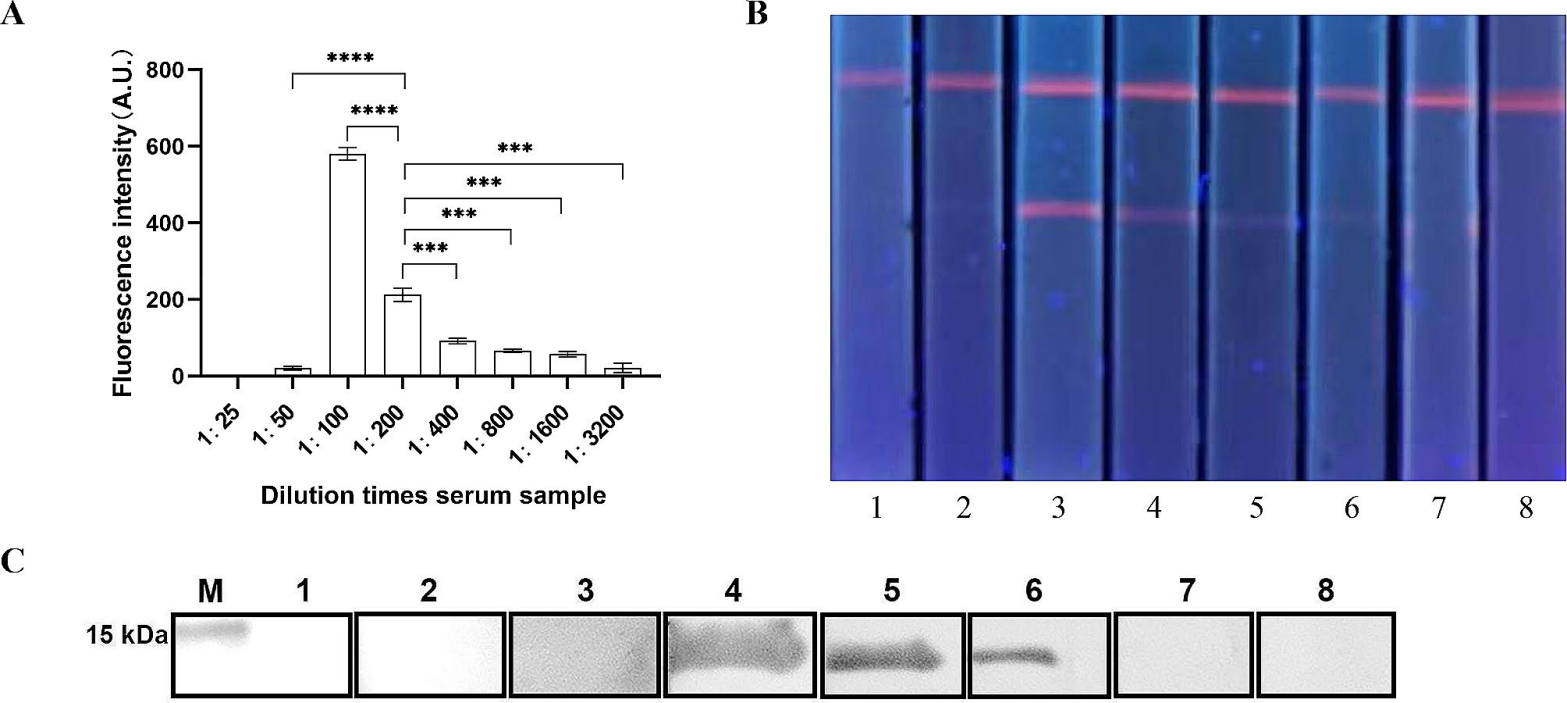
Analytical sensitivity test of ICS. **(A)** A TRF reader was used to analyze ICS signals for different diluted samples. **(B)** Visual results of ICS under ultraviolet light. Lanes 1, 2, 3, 4, 5, 6, 7, and 8 represent the serum samples diluted at 25, 50, 100, 200, 400, 800, 1600, and 3200 times, respectively. ***P < 0.001,****P < 0.0001. **(C)** TSOL18 was analyzed using Western blot with different positive serum dilutions. M: marker. Lanes 1, 2, 3, 4, 5, 6, 7, and 8 represent the serum samples diluted at 25, 50, 100, 200, 400, 800, 1600, and 3200 times, respectively

The serum samples from cysticercosis animals were detected by fluorescence ICS: the fluorescence signal decreased with the increase of serum dilution (Fig. 6). The signal was at its best at 100× dilution, however, even 800× dilution produced a weak signal visible to the naked eye. At 1600 times dilution, the fluorescence intensity of the T line was still higher than the cut off value. Additionally, Western blot analysis was used to validate the analytical sensitivity of TSOL18 (Fig. 6C). The results indicate that specific bands begin to appear when the serum dilution of 1:200, and the bands disappear after 1:800.

### Analytical specificity test

**Fig. 7:**
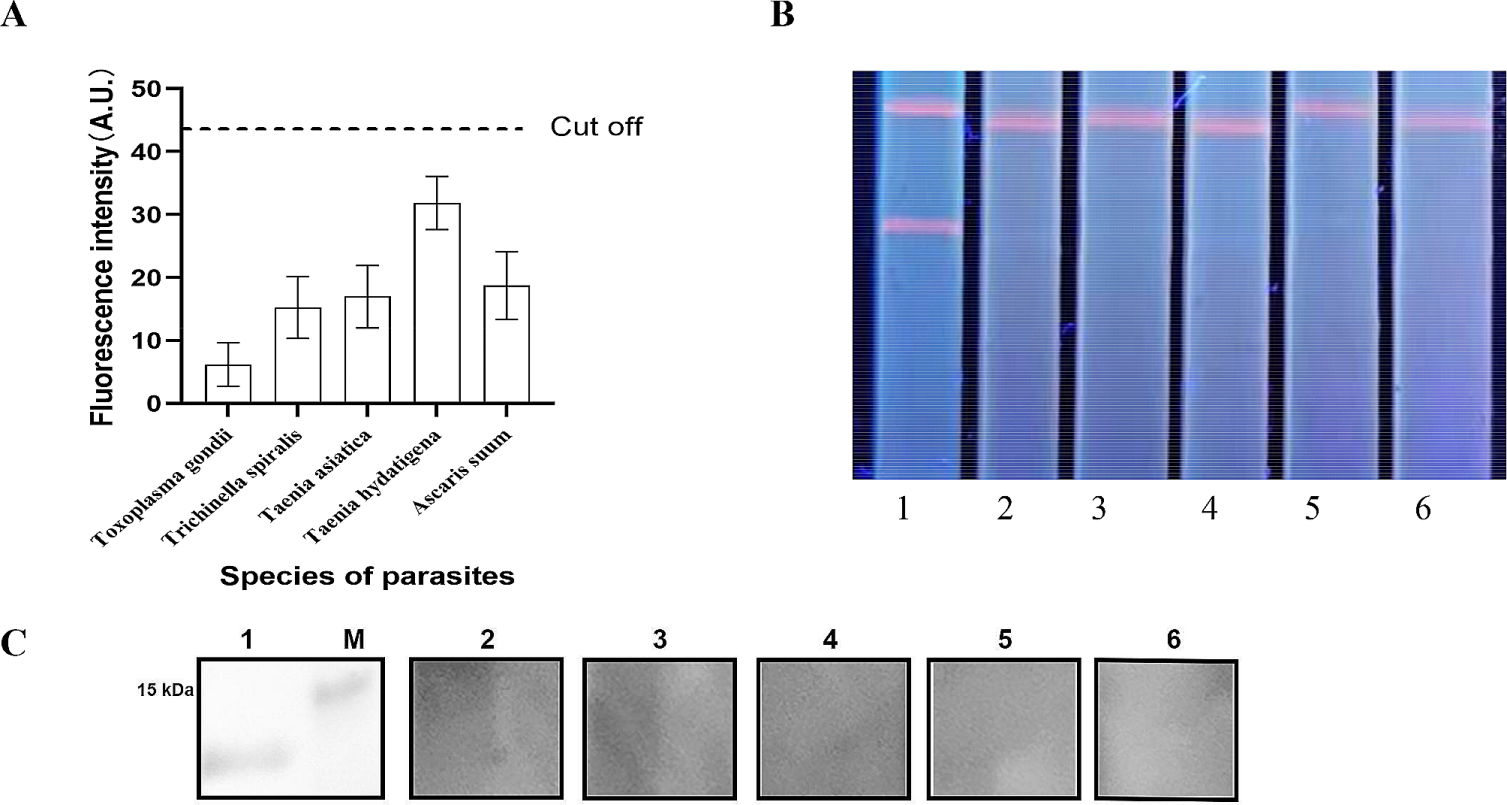
Analytical specificity test of ICS. **(A)** A TRF reader was used to read the ICS signals from different samples. **(B)** Visual results of ICS under ultraviolet light. Lanes 1, 2, 3, 4, 5, and 6 represent positive control and the positive serum samples from *Toxoplasma gondii*, *Trichinella spiralis*, *Taenia asiatica*, *Taenia hydatigena*, and *Ascaris suum*, respectively. **(C)** TSOL18 was analyzed using Western blot with positive serum of different samples. Lanes 1, 2, 3, 4, 5, and 6 represent positive control and the positive serum samples from *Toxoplasma gondii*, *Trichinella spiralis*, *Taenia asiatica*, *Taenia hydatigena*, and *Ascaris suum*, respectively. M: marker

The positive serum samples of *Toxoplasma gondii*, *Trichinella spiralis*, *Taenia asiatica*, *Taenia hydatigena*, and *Ascaris suum* confirmed by ‘gold standard’ were tested by ICS. None of the samples produced a false positive band in ICS, meanwhile, the T-ray fluorescence signal remained below the cut-off value (Fig. 7). This shows that ICS has good specificity and does not detect other similar infections. The analytical specificity test results of ICS were verified by west blot. The results indicate that the two test results were consistent (Fig. 7C).

### Stability test

Test strips good analytical sensitivity and specificity stored at 4 °C for 24 weeks, indicating that they can be stored for at least 6 months at 4 °C. The test strips stored at RT for 16 weeks have good analytical sensitivity and specificity, indicating that they can be stored for up to 4 months at RT. There were no significant differences in color between the detection and control lines. From the 18th week onwards, the color of the T and C lines became lighter, indicating that test strips are potentially good enough for only up to 16 weeks at RT.

### Comparison between ICS and ELISA

**Fig. 8:**
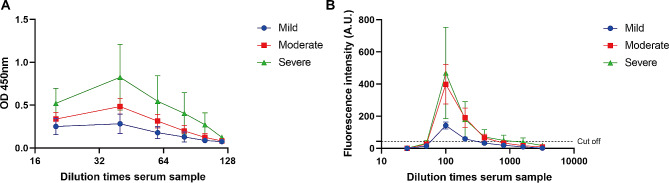
The test results of 105 samples. **(A)** ELISA. **(B)** TRFM-ICS

We used autopsy, ELISA, and ICS methods to test the previously mentioned 133 (105 + 28) samples, and assessed the consistency between the three tests through Cohen’s κ analysis. The 105 samples’ detection results of the three methods were shown (Table 1; Fig. 8). The agreement between autopsy and ICS (*n* = 133) was assessed by Cohen’s κ analysis (Table 2). A highly agreement (κ = 0.925) was observed between the two methods according to κ values reported previously [27]. And the results showed that the proposed ICS are high sensitivity (se: 0.9459) with specificity of 0.9792. The consistency of ICS detection was higher than ELISA, because the diagnostic agreement between ELISA and autopsy was analyzed as 0.870 through Cohen’s κ analysis (Table 3). The Youden descriptor of ICS was 0.9251, so it diagnoses well.

**Table 1:** Comparison of results of different methods

_Infection burden_^Methods^	Necropsy	ICS	ELISA
Negative	68	70	71
Mild(1–20)	7	7	5
Moderate(21–200)	26	25	25
Severe(200+)	4	3	4
Total	105	105	105

**Table 2:** Cohen’s Kappa Statistic for measuring the agreement between Necropsy and ICS in 133 serum samples

Classified by ICS	Classified by Necropsy (as the gold standard)		Total
	T_+_^a^	T_−_ ^b^	
D_+_^c^	35	2	37
D_−_ ^d^	2	94	96
Total	37	96	133
Se^e^	0.9459		
Sp^e^	0.9792		
Jˆ^e^	0.9251		
κ^e^	0.925		

a Samples were tested positive using Necropsy.

b Samples were tested negative using Necropsy.

c Samples were tested positive using ICS.

d Samples were tested negative using ICS.

e The three parameters sensitivity (se), specificity (sp) and Youden index (Jˆ) were calculated according to the Youden descriptor [28]. The κ value was interpreted according to the Landis and Koch descriptors.

**Table 3:** Cohen’s Kappa Statistic for measuring the agreement between Necropsy and ELISA in 133 serum samples

Classified by ELISA	Classified by Necropsy (as the gold standard)		Total
	T_+_^a^	T_−_ ^b^	
D_+_^c^	34	4	38
D_−_ ^d^	3	92	95
Total	37	96	133
κ^e^	0.870		

a Samples were tested positive using Necropsy.

b Samples were tested negative using Necropsy.

c Samples were tested positive using ELISA.

d Samples were tested negative using ELISA.

e The κ value was interpreted according to the Landis and Koch descriptors [27].

## Discussion

At the present time, there are a number of serological tests for porcine cysticercosis that have been published and were originally claimed to offer sensitive and specific diagnosis of porcine cysticercosis, in particular the apDia circulating antigen test developed at the Institute of Tropical Medicine in Antwerp, and an EITB using lentil lectin purified cysticercus glycoproteins developed at the CDC in the US. Despite the original claims for these tests being near perfect in both specificity and sensitivity, both have subsequently been shown to be virtually useless for diagnosis of porcine cysticercosis because of poor specificity - most serologically positive animals have no *T. solium* infection at all [24, 29–31]. The most important failure of all serological tests evaluated to date for porcine cysticercosis is poor specificity and one of the most important causes of cross-reactivity is exposure to, or infection with *Taenia hydatigena*, a ubiquitous taeniid cestode parasite of sheep and goats that also infects pigs [32]. The necropsy is to detect *Cysticercus cellulosae* ’s ‘gold standard’. The only drawback of the overall autopsy is that it is extremely time-consuming and labor-intensive. But it is very time-consuming and labor-intensive, if daily meat inspection relies on autopsies.

There were countless detection methods have been established using ELISA. We can gain a more comprehensive understanding of the benefits of TRFM-ICS by conducting a comparative analysis with ELISA. We used three methods to detect the research samples, using Cohen’s Kappa statistic to determine the consistency of the three methods. The consistency of the diagnosis results between ICS and the gold standard was very high. The se and sq of ICS were 0.9459 and 0.9792 (base on reaserch samples), respectively. This indicates that this ICS has the potential to be further developed. ICS outperforms ELISA in terms of detection speed and the reliability of results (base on the Cohen’s kappa coefficient). ICS also demonstrates significant advantages compared to ELISA in detecting *Trichinella spiralis* [26]. The results indicated that seroconversion of infection pigs was detected by ICS earlier than Qiagen ELISA [26]. Our results showed that the ICS failed to detect two positive samples, one with a moderate infection and one with a severe infection. We have the following explanation for this: the two pigs have already entered the late stage of infection and have not ingested any new eggs, resulting in a very low level of TSOL18 antibodies, which caused the ICS to miss the detection. Because the optimal serum dilution for ELISA is much lower than ICS. We speculate that the antibodies can be detected under this dilution, so the ELISA method did not miss these two samples. The original intention of designing this detection method was to achieve early diagnosis of swine cysticercosis, so that we can treat infected pigs early upon detection. So, it met our expectations when ICS detected all 7 mild pigs.

To minimize the occurrence of multiple infections in the samples, we employed the fecal flotation technique to identify the presence of parasites (such as coccidia, *Ascaris spp.* and *Trichuris spp.*). During the process of dissection, it is essential to confirm the presence of *Taenia hydatigena*, *Taenia asiatica*, or *Trichinella spiralis* infection in the sample. These measures were still unable to completely eliminate the risk of cross-infection with other parasitic diseases (such as *Sarcoptes scabiei* and *Toxoplasma gondii*), because there was a wide range of parasitic diseases in pigs. However, all pathogens that could potentially impact the detection of pig cysticercosis have been eliminated [33]. It is essential to optimize every detail in order to establish an excellent inspection method. The storage buffer utilized for TRFM comprises surfactants and preservatives, which have the potential to influence the coupling efficacy between the microspheres and TSOL18. Therefore, these substances need to be removed by centrifugation before coupling. EDC and NHS are commonly employed as activating reagents in the synthesis of amides to enhance coupling efficiency of carboxylic acids. The determination of the optimal ratio of microspheres to activator and the ratio of EDC to NHS is crucial for the activation of carboxyl groups [34]. Insufficient activation may lead to low coupling efficiency, and decreased sensitivity. Microspheres may aggregate following coupling, primarily due to the disparate isoelectric points of the coupled substances. Therefore, maintaining an appropriate pH value of the coupled system is crucial to ensure the successful outcome of the experiment [34]. The improper pH level can give rise to an uneven distribution of charges, leading to the occurrence of charge settling. The microspheres surface of the unbound target protein still retains the ability to adsorb proteins. Selecting appropriate blocking agents (such as BSA, gelatin, skim milk powder) can effectively reduce subsequent nonspecific binding. The controlled release of microspheres in chromatography plays a critical role, as both excessively rapid or slow release can significantly impact the results [34]. Therefore, the sample material should select the appropriate aperture size and flow rate. There are significant differences between different samples (for example pH and ion strength). Therefore, it is imperative for the sample pad, which serves as the designated area for the droplet-added sample, to possess robust preprocessing and filtering capabilities.

A study found that the gene of TSOL18 runs through the whole life of *Taenia solium*, but the protein expression was limited to the oncosphere lifecycle stage [35]. However, after eating *Taenia solium* eggs, it takes 2–3 months for oncosphere to develop into *Cysticercus cellulosae*, and the method we have established was diagnosed by detecting antibodies in serology [11]. Even if all the oncospheres in pigs developed into cysticercosis, the antibodies in the blood will last for a long time. A prior study used TSOL18 vaccine to immunize young pigs (the maximum interval for immunization were 6 months) determined that the final immune protection rate was as high as 100%. Hence, anti cysticercaus-antibody appeared to remain in the body for at least six months [21]. Moreover, these pigs may often come into contact with parasite eggs again in countries with severe cysticercosis. This further improves the applicable time range of this method.

The sensitivity of the ICS method exhibits 1% increase when compared to the LFA method [22]. Not only does ICS demonstrate exceptional detection results, but it also offers a more cost-effective alternative to LFA. The LFA method requires a small biological sensor to conduct semi-quantitative analysis, and its market price is 290,000 CNY. It is not realistic to equip each testing area with such a device in low- and middle-income countries. Similarly, the market price for the fluorescent quantitative analyzer used for ICS is approximately 5000 CNY. The approximate cost required to produce one ICS (the main materials do not exceed 1 CNY). The cost of ICS was not high, and could be lower with mass production. Excessively humid environments and excessively strong light may be detrimental to the storage of ICS, considering different application scenarios in the real world. However, purchasing aluminum foil vacuum bags and a household vacuum sealer can help store ICS effectively. The detection process of ICS is very easy. 1 µl of serum was added to a pre-prepared buffer, mixed, and then dropped into the sample loading area. The results can be determined by using a portable UV lamp for illumination after waiting. Ordinary individuals can also readily accomplish the testing. So, this ICS fully caters to market demands and is equally practical in various real-world environments.

There was no cross-reaction to *Taenia hydatigena* in our ICS method. However, no literature on *Taenia hydatigena* and TSOL18 has been published, and the theoretical reason is not yet clear. We speculate that the oncosphere larval stage of the *Taenia hydatigena* lacks antigens similar to TSOL18. One of our objectives is to collect the eggs and comprehensively examine the factors contributing to this phenomenon through experimental analysis in the future. The ICS method is currently limited to laboratory testing with sample size of 105 samples. We plan to collect pig meat and serum samples (at least 1000 samples) in the high-prevalence areas of porcine cysticercosis (at least three locations). The pork samples are subjected to necropsy, and the serum samples are analyzed using the ICS method in order to further investigate its practicality. The development of ICS using whole blood as a sample would be a new breakthrough, because the preparation of serum from whole blood still requires pre-processing. The method has not been tested on human cysticercosis patients. It would be a significant breakthrough if this product can be applied to humans. If these two problems can be resolved, it may be possible to achieve quantitative analysis of *Cysticercus cellulosae*. Firstly, the minimum detectable level of TSOL18 antibodies in the body. Secondly, the level of TSOL18 antibodies changes caused by the parasitic infection of one or more *Cysticercus cellulosae*. Can we predict the number of cysts in this pig by measuring the T-line fluorescence intensity? The improvement of detection techniques for cysticercosis remains a significant challenge that requires further advancements.

## Conclusions

The preliminary evaluation of the TRFM-ICS method for detecting *Cysticercus cellulosae* by detecting anti-TSOL18 serological antibody indicates that the test results are good in analytical sensitivity and specificity.Further testing is required on a large number of experimental samples ultimately in equal number of true positive and negative samples for precise validation to evaluate diagnostic sensitivity, specificity and accuracy.

### Electronic supplementary material

Below is the link to the electronic supplementary material.

Supplementary Material 1

## Data Availability

All data generated or analyzed during this study are included in this published article. Further inquiries can be directed to the corresponding authors.

## Declarations

### Statement approved by the institutional animal care and use committee

This study carried out the principles of laboratory animal affairs management in China. The experiment was approved by the Animal Protection and Use Committee of Jilin University (20170318). All experiments were performed in accordance with relevant guidelines and regulations.

## Abbreviations

ICS: immunofluorescence chromatography test strip
TRFM: time-resolved fluorescent microspheres
ELISA: indirect enzyme-linked immunosorbent assay
Dot-ABC-ELISA: dot avidin-biotin complex enzyme-linked immunosorbent assay
TRFM-ICST: Time-resolved fluorescence microsphere immunochromatography strip test
RT: room temperature
EITB: enzyme-linked immunoelectrotransfer blot
