# Supplementary material for: Development and evaluation of time-resolved rapid immunofluorescence test for detection of TSOL18 specific antibody in porcine cysticercosis infections
Source: BMC Vet Res. 2024 May 8;20:182. doi: 10.1186/s12917-024-04034-7 (PMC11077887; doi:10.1186/s12917-024-04034-7)
Supplement: Supplementary file 1 — Supplementary Material 1 [file 12917_2024_4034_MOESM1_ESM.pdf]

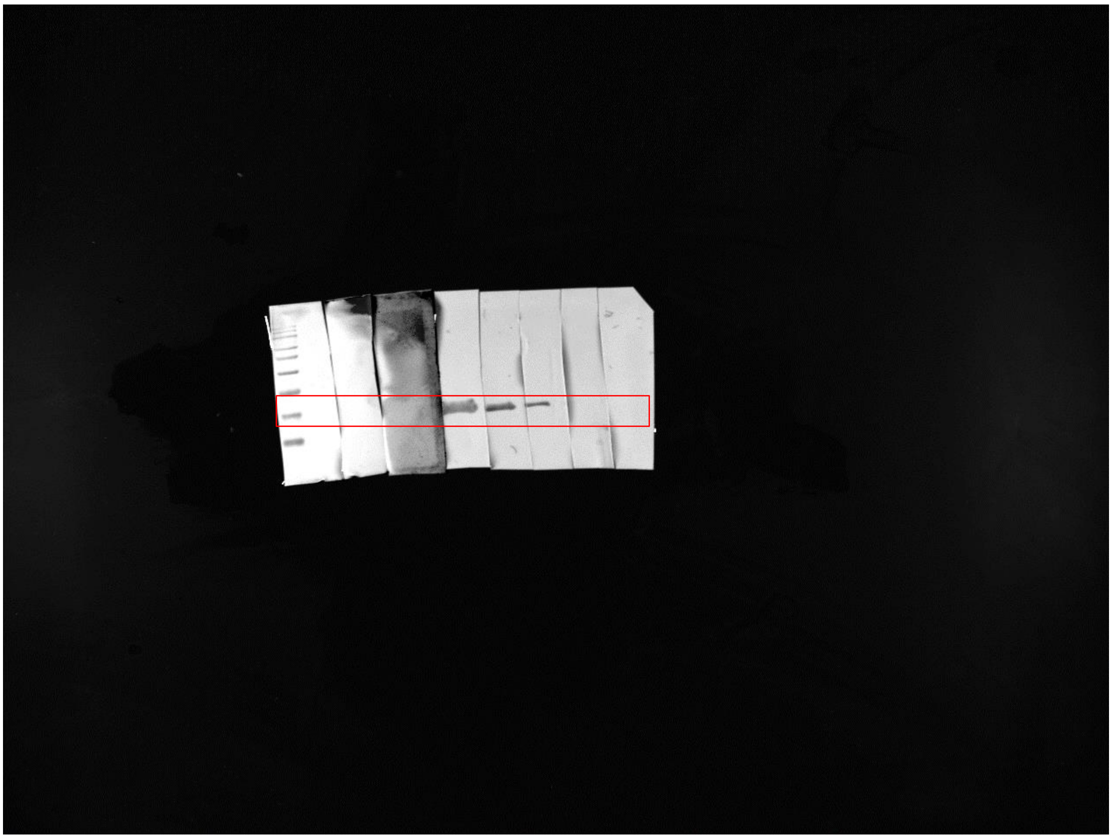

Figure 6C. TSOL18 was analyzed using Western blot with different positive serum dilutions. M: marker. Lanes 1, 2, 3, 4, 5, 6, 7, and 8 represent the serum samples diluted at 25, 50, 100, 200, 400, 800, 1600, and 3200 times, respectively.

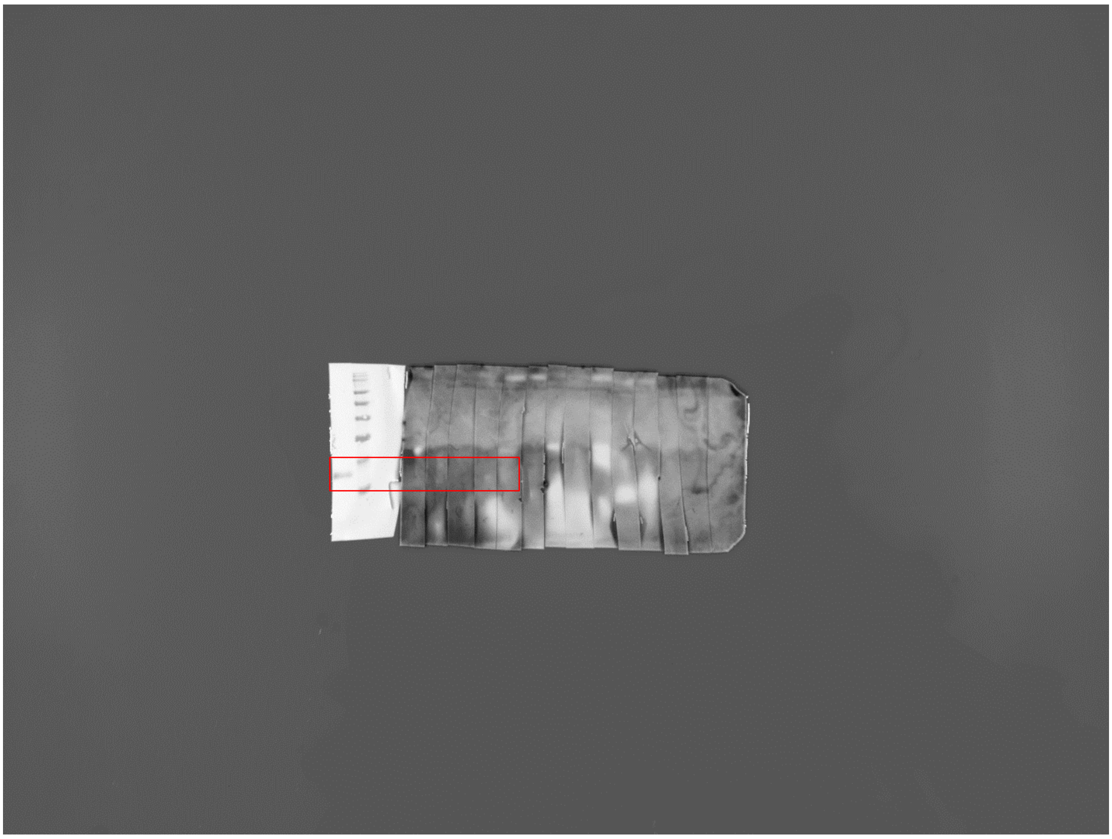

Figure 7C. TSOL18 was analyzed using Western blot with positive serum of different samples.

Lanes 1, 2, 3, 4, 5, and 6 represent positive control and the positive serum samples from Toxoplasma gondii, Trichinella spiralis, Taenia asiatica, Taenia hydatigena, and Ascaris suum, respectively. M: marker.
